# Association of Medicaid expansion with birth outcomes: evidence from a natural experiment in Texas

**DOI:** 10.1186/s12889-024-19007-6

**Published:** 2024-06-03

**Authors:** Meryem Saygili, Esra Eren Bayindir

**Affiliations:** 1 University of Texas at Tyler, Tyler, TX 75799 USA; 2 Hamburg Center for Health Economics (HCHE), University of Hamburg, Esplanade 36, 20354 Hamburg, Germany

**Keywords:** Medicaid expansion, Adverse birth outcomes, Race/ethnicity, Texas

## Abstract

**Background:**

Empirical evidence on the effects of Medicaid expansion is mixed and highly state-dependent. The objective of this study is to examine the association of Medicaid expansion with preterm birth and low birth weight, which are linked to a higher risk of infant mortality and chronic health conditions throughout life, providing evidence from a non-expansion state, overall and by race/ethnicity.

**Methods:**

We used the newborn patient records obtained from Texas Public Use Data Files from 2010 to 2019 for hospitals in Texarkana, which is located on the border of Texas and Arkansas, with all of the hospitals serving pregnancy and childbirth patients on the Texas side of the border. We employed difference-in-differences models to estimate the effect of Medicaid expansion on birth outcomes (preterm birth and low birth weight) overall and by race/ethnicity. Newborns from Arkansas (expanded Medicaid in 2014) constituted the treatment group, while those from Texas (did not adopt the expansion) were the control group. We utilized a difference-in-differences event study framework to examine the gradual impact of the Medicaid expansion on birth outcomes.

**Results:**

Medicaid expansion was associated with a 1.38-percentage-point decrease (95% confidence interval (CI), 0.09–2.67) in preterm birth overall. Event study results suggest that preterm births decreased gradually over time. Medicaid expansion was associated with a 2.04-percentage-point decrease (95% CI, 0.24–3.85) in preterm birth and a 1.75-percentage-point decrease (95% CI, 0.42–3.08) in low birth weight for White infants. However, Medicaid expansion was not associated with significant changes in birth outcomes for other race/ethnicity groups.

**Conclusions:**

Our findings suggest that Medicaid expansion in Texas can potentially improve birth outcomes. However, bridging racial disparities in birth outcomes might require further efforts such as promoting preconception and prenatal care, especially among the Black population.

**Supplementary Information:**

The online version contains supplementary material available at 10.1186/s12889-024-19007-6.

## Background

The state-level Medicaid expansions play a central role in the Affordable Care Act (ACA) and are intended to increase access to health care and improve health outcomes. Texas is one of the few states that did not expand Medicaid as of 2024, while all its adjacent states, including Arkansas, did so. Arkansas expanded Medicaid through a Sect. 1115 waiver in 2014. This approach, known as the “private option”, uses Medicaid funds to purchase private health plans on the state’s marketplace. A two-state city, Texarkana, represents an unusual example where the poor living in the Arkansas half of town gained access to the benefits of the Medicaid expansion but nothing changed for those on the Texas side. This study aims to explore the effect of this expansion on birth outcomes both overall and by race/ethnicity.

Preterm birth leads to important long-term impacts on health outcomes both during childhood and afterward. It is the most frequent cause of infant death in the US, accounting for over one-third of infant deaths [1], and it is a predictor of psychiatric morbidity [2]. Given the large gaps in the use of preventive care between Medicaid/uninsured and commercially insured women [3], extending health insurance coverage to the uninsured population may lead to better prenatal health and improve population health outcomes [4–6].

When first introduced, access to Medicaid at the time of birth improved infants’ access to life-saving hospital services [7, 8]. Moreover, Medicaid expansions from 1997–2009 reduced severe psychological distress in low-income parents [9, 10]. Even though the Medicaid expansion of 2014, which increased income eligibility to 138% of the federal poverty level (FPL) for all adults, did not influence the eligibility threshold for pregnant women (which was 200% of the FPL in both Arkansas and Texas before the expansion), it led to detectable improvements in some preconception health measures [11] and increased continuity of insurance coverage and access to postpartum care. [12, 13]^1^ The expansion of Medicaid in Arkansas was associated with a significant increase in continuous insurance coverage and outpatient visits during the first 6 months postpartum [14]. By improving insurance continuity in the perinatal period for low-income women, Medicaid expansion might help improve the quality of perinatal health care, which is more likely to be realized in the long term [13, 15, 16].

Even though better health outcomes for newborns might be viable due to improved access to preconception or prenatal care and reduced out-of-pocket spending, evidence on the effect of Medicaid expansion on birth outcomes is mixed. Several studies find no significant effect of expansion on birth outcomes [17–21], which are usually attributed to already good coverage during pregnancy prior to policy interventions. However, expanding Medicaid coverage had positive effects on low birth weight and preterm birth for low-income households in Oregon [22].

Most of the empirical work finds no significant effect of Medicaid expansion on birth outcomes in general, with mixed evidence on the impact of extended coverage on racial/ethnic disparities [23], which are abundant in birth outcomes. In 2019, 9.26% of White babies were preterm, whereas 14.39% of Black, and 9.97% of Hispanic babies were preterm, and 6.89% of White, 14.15% of Black, and 7.55% of Hispanic infants had low birth weight in the US [24]. County-level variation declined for low birth weight and preterm birth among all racial/ethnic categories suggesting improved equity in expansion states [25]. Even though Medicaid expansion was not significantly associated with differences in rates of low birth weight or preterm birth outcomes, it led to improvements for Black infants [26]. Infant mortality rate decline was greater in Medicaid expansion states, with greater declines among Black infants (in states and Washington, DC) [27]. However, another study found that infant mortality decreased for babies born to White mothers as a result of Medicaid expansion, but not for babies born to Black mothers [28]. Therefore, the results seem to be sensitive to sample selection, which makes the external validity of the findings questionable.

Medicaid expansion of 2014 was more likely to be adopted in states where policymakers of the time prioritized health policies. However, those states with health policies high on the agenda were presumably more likely to launch programs to improve population health, increase the funding of social services, and provide support to health care services than the non-expansion states, which might also result in an improvement in health outcomes. Hence, it is difficult to isolate the impact of Medicaid expansion on health outcomes in general. However, in our case, the Arkansan population residing in Texarkana gained access to Medicaid expansion, with being subject to the hospital care of a non-expansion state. Hence, the association between Medicaid expansion and birth outcomes in Texarkana would presumably be due to Medicaid expansion, not some other measures taken concurrently with Medicaid expansion to improve the quality of health care services. Moreover, even though Arkansas was self-selected into Medicaid expansion because local policymakers decided to prioritize health policies generally around 2014, healthcare needs and health outcomes of the Arkansan population in Texarkana presumably did not have a major effect on the state’s Medicaid expansion decision. Hence, about half of the population in Texarkana gained access to Medicaid expansion in 2014 while they continued to utilize the hospitals on the Texas side of Texarkana. Therefore, this natural experiment provides a unique opportunity to examine how the birth outcomes would change as a result of Medicaid expansion with no related changes on the provider side.

This paper examines if the Medicaid expansion was associated with changes in rates of preterm birth and low birth weight overall and by race/ethnicity in Texas hospitals located in Texarkana, where half of the population lives in Arkansas and all of the hospitals serving obstetrics patients are located in Texas. We conduct a difference-in-differences analysis where newborns from Arkansas that expanded Medicaid in 2014 constitute the treatment group, while those from Texas are the control group. By examining the association of Medicaid expansion with birth outcomes using a natural experiment from Texas, a non-expansion state, we provide evidence on potential effects of an increase in health care coverage on preterm birth and low birth weight.

## Methods

### Data

We used newborn inpatient records from singleton births that occurred in hospitals located within Texarkana, Texas, from 2010 to 2019. The data are from the Texas Department of State Health Services Hospital Discharge Database, which collects hospital discharge data from all state licensed hospitals [29]. Data are available by quarter. This study was exempt from Institutional Review Board review because data were anonymized and publicly available. The hospitals located on the Texas side serve patients from both the Texas and Arkansas side of the city. The data capture in-hospital newborn records accurately since all hospitals are on the Texas side (except for a behavioral health hospital, which is irrelevant to our study). There are 25,220 singleton birth records over the ten years, with an average of 2,522 admissions per year.

### Outcome and control variables

Outcome variables are preterm birth (< 37 weeks gestation) and low birth weight (< 2500 g). International Statistical Classification of Diseases and Related Health Problems (ICD) codes used to identify birth outcomes are reported in Additional file 1.

In the difference-in-differences framework, it is not necessary to control for time-invariant cofounders but it is important to control for time-varying cofounders. Hence, we included controls for potentially time-varying cofounders. The independent variables are race/ethnicity and sex categories. We construct race/ethnicity categories using race and ethnicity variables reported in the dataset. The mutually exclusive categories are non-Hispanic White (if the race is White and ethnicity is non-Hispanic, hereafter “White”); non-Hispanic Black (if the race is Black and ethnicity is non-Hispanic, hereafter “Black”); Hispanic (if ethnicity is Hispanic); and other (all other categories). The newborns’ sex (female and male) is also available in the dataset.

The data report the zip codes for the patients’ residences. In all regressions, we include dummies for patient zip codes as a proxy for time-invariant socioeconomic characteristics of patient localities within Arkansas and Texas sides of Texarkana to account for possible changes in the geographical composition of births. We also include dummies for year-quarters to control for factors that are constant across entities but vary over time.

### Statistical analysis

To assess whether birth outcomes differed between infants from Texas and Arkansas before and after the Medicaid expansion, we used a multivariable difference-in-differences model adjusted for sex, race/ethnicity, patient zip code, and year-quarter dummies. The treatment group includes newborns from Arkansas, while the control group includes those from Texas. The Medicaid expansion became effective on January 1, 2014, in Arkansas. Thus, the pre-expansion period is 2010–2013, and the post-expansion period is 2014–2019. Because we observe all of the population of interest rather than a random sample, we used the heteroscedasticity robust standard errors following Abadie et al. (2023) [30].

To examine heterogeneity in the association between Medicaid expansion and birth outcomes by race/ethnicity, we estimated the difference-in-differences model for each race/ethnicity group separately. We also employed a difference-in-difference-in-differences model to explore if the expansion affected different racial/ethnic groups differently.

Even though Medicaid expansion does not influence the eligibility threshold for pregnant women, it improves insurance continuity in the perinatal period for low-income women, and thus, might improve the quality of perinatal health care and birth outcomes. Its impact is more likely to be realized in the long term. Therefore, we conducted a difference-in-differences event-study analysis to break down the effect over the years.

The validity of the difference-in-differences analysis hinges on the assumption that the difference in birth outcomes between the infants from Texas and Arkansas would have remained constant over time if Medicaid expansion in Arkansas had not taken place (parallel trends assumption). We found no evidence that the parallel trends assumption is violated (see Additional file 2 for details on the statistical analysis).

The dataset does not allow us to match the mothers and infants. Hence, we cannot control for mother characteristics in the analysis. However, we examined characteristics of pregnancy, childbirth, and the puerperium patients discharged from Texarkana hospitals during the same period. We analyzed the association between the comorbidities of mothers, measured by the Charlson Comorbidity Index and Medicaid expansion considering that Medicaid expansion is positively associated with the incidence of gestational diabetes [31]. We also examined the distribution of childbirth patients into different age groups and payer sources by race/ethnicity (see Additional file 3 for details).

## Results

Table 1 shows the means of variables by the state in the pre-expansion period as well as the t-test results showing whether the differences between the states are statistically significant. 50.96% of newborns reside on the Texas side of Texarkana. In terms of race, the share of White newborns is similar. However, Texas has more Black newborns, while Arkansas has more Hispanic newborns. The percentage of female newborns from Arkansas and Texas is not statistically significantly different. 6.63% of infants were preterm, whereas 4.58% had low birth weight prior to 2014 in Texarkana. Averages of outcome variables are not statistically significantly different across the states in the pre-expansion period.

**Table 1:** Individual characteristics and outcome variables by the state in the pre-expansion period

	Arkansas	Texas	Texas-Arkansas	*p*-values
Patient share (%)	49.07	50.96	1.89^***^	< 0.01
Race/ethnicity (%)				
Non-Hispanic White	53.61	54.42	0.82	0.41
Non-Hispanic Black	23.98	29.52	5.54^***^	< 0.01
Hispanic	9.00	3.68	-5.32^***^	< 0.01
Other	13.41	12.37	-1.03	0.12
Sex (%)				
Female	48.86	48.17	-0.70	0.48
Outcome variables (%)				
Preterm	6.89	6.37	-0.52	0.29
Low birth weight	4.50	4.66	0.16	0.70
Observations	5,020	5,213		

Arkansas (expanded Medicaid in 2014). Texas (did not expand Medicaid). The pre-expansion period refers to 2010–2013. The “Other” race/ethnicity category includes all records not classified as non-Hispanic White, non-Hispanic Black, or Hispanic. The table reports the averages of individual characteristics in each state, and whether the difference is statistically significant based on a t-test. *P*-values of t-tests comparing means for Texas and Arkansas are reported in the last column

^***^p < 0.01

Table 2 shows the averages of outcome variables for different race/ethnicity groups in both states before the Medicaid expansion. Black newborns were more likely to be preterm and have low birth weight than White newborns in both states, while the difference is statistically insignificant for preterm infants in Arkansas. However, there were no significant differences between White and Hispanic newborns in birth outcomes in either state.

**Table 2:** Outcome variables by race/ethnicity in the pre-expansion period

	Black	White	Hispanic	(White-Black)	*p*-values of t-test WB	(White-Hispanic)	*p*-values of t-test WH
Arkansas (%)							
Preterm	7.81	6.65	4.65	-1.16	0.19	2.01	0.11
Low birth weight	6.98	3.57	3.10	-3.41***	< 0.01	0.47	0.62
Observations	1,204	2,691	452				
Texas (%)							
Preterm	7.86	5.92	4.17	-1.94**	0.014	1.76	0.31
Low birth weight	7.73	3.38	3.13	-4.35***	< 0.01	0.26	0.85
Observations	1,539	2,837	192				

The averages of outcomes variables in each race/ethnicity category and whether the differences are statistically significant based on t-tests are reported. t-tests WB and WH refer to the t-test comparing the means of White and Black, and White and Hispanic, respectively. Black, non-Hispanic Black; White, non-Hispanic White. The pre-expansion period refers to 2010–2013

^***^p < 0.01, **p < 0.05

Table 3 shows the unadjusted and adjusted changes in birth outcomes associated with Medicaid expansion, overall and by race/ethnicity. We find that Medicaid expansion is associated with a 1.38 percentage points decrease (95% CI, 0.09–2.67) in preterm birth overall. Considering the difference-in-differences estimates separately for White, Black, and Hispanic infants, the results indicate that the Medicaid expansion is associated with a 2.04 percentage points decrease (95% CI, 0.24–3.85) in preterm rate and 1.75 percentage points decrease (95% CI, 0.42–3.08) in low birth weight rate for White infants. Although statistically insignificant, Medicaid expansion is associated with a decline in the preterm birth rate for Black infants. Results for low birth weight rates for Black infants and birth outcomes for Hispanic infants are insignificant. The last column shows difference-in-difference-in-differences estimates where White infants are the baseline category. The coefficients capture if the expansion had a differential effect for the corresponding race category compared to White infants. We do not find any statistically significant relationship between Medicaid expansion and racial/ethnic disparities in birth outcomes.

**Table 3:** Unadjusted and adjusted changes in birth outcomes associated with Medicaid expansion

	Arkansas		Texas		DID		DDD
	Pre-	Post-	Pre-	Post-	Unadjusted (95% CI)	Adjusted (95% CI)	Adjusted (95% CI)
Overall							
Preterm	6.89	6.97	6.37	7.67	-1.22* (-2.51, 0.06)	-1.38** (-2.67, -0.09)	
Low birth weight	4.50	4.70	4.66	5.52	-0.66 (-1.74, 0.43)	-0.80 (-1.88, 0.29)	
Non-Hispanic White							
Preterm	6.65	6.76	5.92	8.09	-2.06** (-3.85, -0.26)	-2.04** (-1.96, -0.17)	
Low birth weight	3.57	2.94	3.38	4.48	-1.73*** (-3.05, -0.41)	-1.75*** (-3.08, -0.42)	
Non-Hispanic Black							
Preterm	7.81	8.84	7.86	9.88	-0.99 (-3.76, 1.78)	-1.22 (-4.02, 1.57)	1.12 (-2.09, 4.32)
Low birth weight	6.98	8.36	7.73	8.76	0.35 (-2.32, 3.03)	0.35 (-2.35, 3.05)	2.10 (-0.58, 4.77)
Hispanic							
Preterm	4.65	5.55	4.17	4.30	0.77 (-3.22, 4.76)	-0.37 (-4.63, 3.88)	1.96 (-3.00, 6.91)
Low birth weight	3.10	4.40	3.13	3.90	0.53 (-3.08, 4.15)	0.77 (-3.07, 4.61)	2.07 (-1.72, 5.87)

The estimates are multiplied by 100 to provide percentage-point differences. The adjusted estimates are adjusted for sex, year-quarter, and patient zip code dummies. Dummies for race/ethnicity categories are also included in the DID model for the overall results. The DID regressions for race/ethnicity categories only include infants in the corresponding race/ethnicity categories. In the DDD regressions, non-Hispanic White infants are the baseline group. The DDD regressions include infants in the corresponding race/ethnicity category and the baseline group of non-Hispanic White infants

*DID* difference-in-differences, *DDD* difference-in-difference-in-differences, *Pre-* pre-Medicaid expansion period (2010–2013), *Post-* post-Medicaid expansion period (2014–2019), *CI* confidence interval

^***^p < 0.01, **p < 0.05, *p < 0.1

**Fig. 1:**
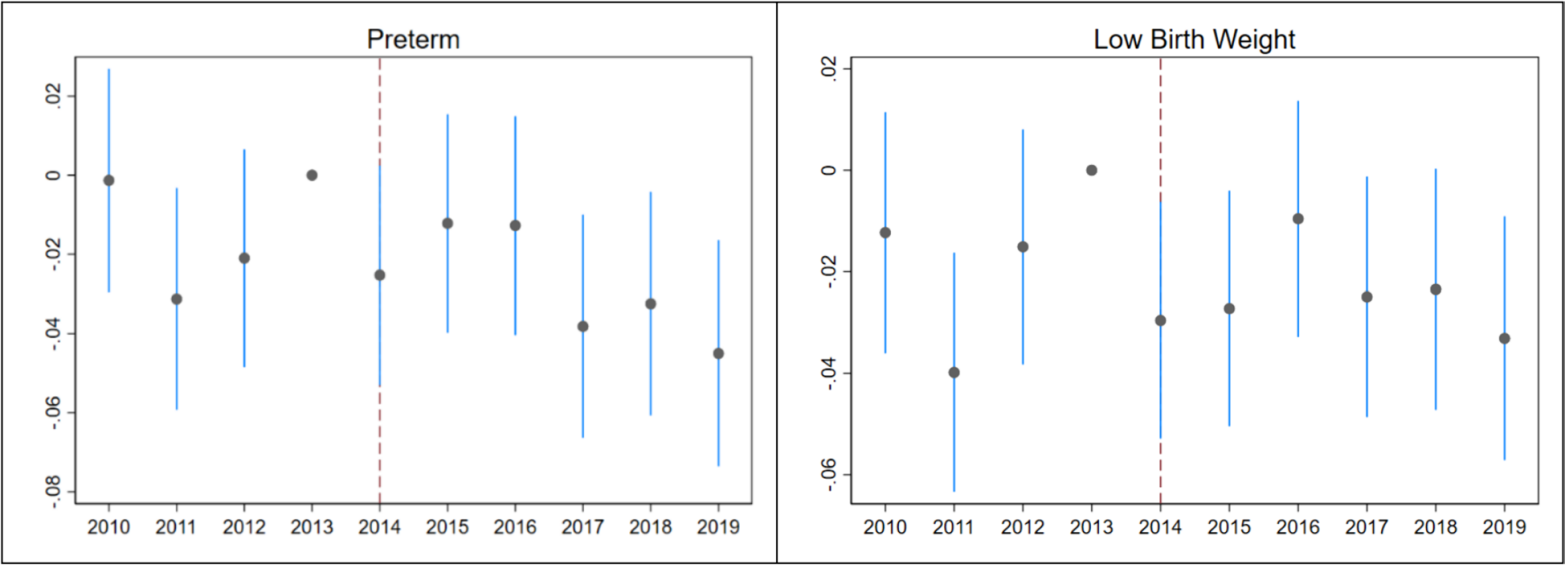
Difference-in-differences event study results. The coefficient estimates with 95% confidence intervals show the differences in outcomes between the treatment and control groups over the years. 2013 is the reference year

Difference-in-differences event study results are shown in Fig. 1. Medicaid expansion is associated with larger declines in preterm birth over time, whereas there seems to be an instantaneous decline in low birth weight, which is preserved over time.

Supplemental Table 1 in Additional file 2 reports the regression results for testing parallel trends assumption. We fail to reject that the time trends for birth outcomes of newborns from the Texas and Arkansas sides of Texarkana were different in the pre-expansion period at the 5% level. Hence, we find no evidence that the parallel trends assumption is violated, supporting the validity of the difference-in-differences analysis.

To identify possible explanations as to why Medicaid expansion may have affected different racial/ethnicity groups differently, we analyzed childbirth patients. Supplemental Table 2 in Additional Files 3 reports unadjusted and adjusted changes in mothers’ comorbidities associated with Medicaid expansion. Even though the average comorbidity index for Black mothers has risen in Arkansas compared to those in Texas, we did not find a statistically significant association.

Additional File 3 Supplemental Table 3 reports the age distribution of mothers who gave birth in Texarkana hospitals in the pre-expansion and post-expansion periods by state and race. For White mothers, the share of teenage pregnancies declined in both Arkansas and Texas from the pre-expansion period to the post-expansion period, with a larger decline in Arkansas than in Texas; the share of White mothers with years of age from 20 to 34 increased whereas there was no statistically significant change in the share of mothers with age 35 and over from pre-expansion period to post-expansion period in Arkansas; share of White mothers with age 35 and over increased from pre-expansion period to post-expansion period in Texas. For Black mothers, the share of teenage pregnancies declined from the pre-expansion period to the post-expansion period in both Arkansas and Texas, with a larger decline in Texas than that of Arkansas. There was no statistically significant change in the share of Black mothers aged 20–34 from the pre-expansion period to the post-expansion period in Arkansas, while this share increased in Texas. Insurance type distribution of mothers who gave birth in Texarkana hospitals in the pre-expansion and post-expansion period by state and race is reported in Additional File 3 Supplemental Table 4. The share of privately insured White mothers increased by 2.68 percentage points from pre-expansion to post-expansion, while the share of uninsured White mothers decreased by 1.6 percentage points from pre-expansion to post-expansion in Arkansas. For White mothers in Texas, the share of privately insured declined by 3 percentage points, while the share of mothers covered by Medicaid increased by 3.06 percentage points from the pre-expansion period to the post-expansion period. While the direction of change from pre-expansion period to post-expansion period in Medicaid and privately insured was the same for Black mothers as White mothers in both Arkansas and Texas, the changes in the shares from pre-expansion period to post-expansion period by insurance type were statistically insignificant for Black mothers in both states.

## Discussion

In this work, we examined if the Medicaid expansion was associated with changes in rates of preterm birth and low birth weight, both overall and by race/ethnicity, in Texas hospitals located in Texarkana, where half of the population lives in Arkansas but all of the hospitals serving obstetrics patients are in Texas. Therefore, we provide evidence from hospitals in Texas, a non-expansion state, for the effect of a potential Medicaid expansion on birth outcomes. We find that Medicaid expansion is statistically significantly associated with a decline in preterm birth rate overall and declines in adverse birth outcomes for White infants.

Medicaid expansion was associated with improvements in birth outcomes overall. However, in contrast with most of the studies examining the effect of Medicaid expansion on racial disparities [25–27] and in line with Cook and Stype [28], we find that Medicaid expansion is associated with better outcomes for White newborns but fail to find a significant relationship between Medicaid expansion and birth outcomes for Black newborns in Texarkana.

To find a possible explanation for the lack of a statistically significant association between Medicaid expansion and birth outcomes for Black infants, we examined the association between comorbidities of mothers, measured by the Charlson Comorbidity Index, and Medicaid expansion in Texarkana hospitals. Even though the average comorbidity index for Black mothers has risen in Arkansas compared to those in Texas, we did not find a statistically significant association. No significant improvements in birth outcomes for Black babies might also be the result of the trend toward older maternal age, which affects the birth outcomes of Black women disproportionately causing an increase in racial inequity in birth outcomes [32]. We examined the age distribution of mothers in the pre-expansion and post-expansion periods in the Arkansas and Texas sides of Texarkana. The change in the age distribution of the White (Black) mothers from the pre-expansion period to the post-expansion period is expected to lead to bigger (smaller) improvements in birth outcomes in Arkansas than in Texas. Hence, the changes in the age distribution of mothers might partly explain our findings. Moreover, the fact that there was no decline in the uninsured rate of Black mothers from the pre-expansion period to the post-expansion period residing in Arkansas side of Texarkana suggests that White mothers rather than the Black mothers were the main beneficiaries of the Medicaid expansion with the private option in Arkansas, which might be partly driving our findings.

Our findings rely on the assumption that there were no other changes taking place concurrently with Medicaid expansion in Arkansas or Texas that would affect the birth outcomes in Texarkana hospitals. To the best of our knowledge, there were no maternal health campaigns, changes in food or housing programs, and changes in the health care providers serving obstetrics patients in Texarkana, that would bias our findings.

Our study has several limitations. First, we exploit a natural experiment in Texarkana to estimate the effect of a possible Medicaid expansion controlling for the factors our dataset allows and our results are mainly valid for Texarkana. Second, in our analysis, we consider the hospitals in Texarkana, Texas; however, if patients of a certain race/ethnicity who are more likely to have adverse birth outcomes are more likely to go to hospitals outside Texarkana, the validity of our results will be questionable. We have examined the newborns residing in Texarkana and born in other Texas hospitals; there is no statistically significant difference in birth outcomes pre-expansion and post-expansion by race/ethnicity and state. We do not observe the patients residing in Texarkana but going to hospitals outside Texas. However, the hospitals within 50 miles of distance on the Arkansas side are rural hospitals much smaller than the hospitals in Texarkana. Moreover, with neonatal facilities with level III (neonatal intensive care) and level II (special care) care, hospitals in Texarkana are well-equipped for complicated deliveries. Therefore, mothers with risky pregnancies are unlikely to go to the surrounding healthcare facilities in Arkansas for delivery. Third, given that Arkansas expanded Medicaid via private option rather than traditional Medicaid expansion, the findings may not apply in the case of traditional Medicaid expansion. However, studies found similar conclusions regarding the effects of the private option and traditional Medicaid expansions [33, 34]. Finally, there have been some disruptions that adversely affected insurance continuity in Arkansas that may cause us to underestimate the impact of the Medicaid expansion [35].

## Conclusions

The main contribution of our work is providing evidence for the effect of a potential Medicaid expansion on birth outcomes from a non-expansion state. Even though ACA Medicaid expansion does not primarily target improving birth outcomes, we find evidence from Texarkana that Medicaid expansion is statistically significantly associated with a decrease in overall preterm birth rate; however, we fail to find a statistically significant effect of Medicaid expansion on racial disparities in birth outcomes. Hence, if Texas expands Medicaid, bridging racial disparities in birth outcomes might require further efforts and policies such as promoting preconception and prenatal care, especially among the Black population to reduce the disparities between Black and White infants.

### Supplementary Information

Supplementary Material 1.

## Data Availability

Datasets analyzed in this study are available from the Texas Department of State Health Services: https://www.dshs.texas.gov/thcic/hospitals/Inpatientpudf.shtm and code to replicate this study is available upon request.

## Abbreviations

ACA: Affordable Care Act
ICD: International Statistical Classification of Diseases and Related Health Problems

## Footnotes

1 Even though Texas did not adopt Medicaid expansion, there have been some efforts to increase insurance continuity for women with Medicaid. Texas launched the Healthy Texas Women program on July 1, 2016, which provides women's health and family planning services for low-income Texas women besides the Medicaid for Pregnant Women and CHIP Perinatal programs. However, it takes time until such programs get publicized, and only recently (in 2020), the program was expanded to provide prenatal care and gained more publicity through the demonstration waiver granted by the Centers for Medicare & Medicaid Services. Even though the program is significant, it is unlikely to affect the current study that spans years 2010 to 2019.
