# Supplementary material for: Association of Medicaid expansion with birth outcomes: evidence from a natural experiment in Texas
Source: BMC Public Health. 2024 Jun 3;24:1486. doi: 10.1186/s12889-024-19007-6 (PMC11149325; doi:10.1186/s12889-024-19007-6)
Supplement: Supplementary file 1 — Supplementary Material 1. [file 12889_2024_19007_MOESM1_ESM.docx]

**Additional File 1**

**Outcome Variables**

We use the International Classification of Diseases (ICD) codes to construct our outcome variables. The data report ICD-9 codes until the third quarter of 2015 and ICD-10 afterward. First, we keep single live-born only. We retain discharges if the principle or (up to 24) secondary diagnoses contain ICD-9 code of V30 or ICD-10 codes of Z3800, Z3801, or Z381. Preterm, an indicator for preterm birth, is a binary variable that takes the value of one if the principle or secondary diagnoses code include ICD-9 codes of 76521, 76522, 76523, 76524, 76525, 76526, 76527, 76528 or ICD-10 codes of P072, P0720, P0721, P0722, P0723, P0724, P0725, P0726, P073, P0730, P0731, P0732, P0733, P0734, P0735, P0736, P0737, P0738, P0739. Low birth weight is also a binary variable that takes the value of one if the principal or secondary diagnoses include the ICD-9 codes of 76401, 76402, 76403, 76404, 76405, 76406, 76407, 76408, 76411, 76412, 76413, 76414, 76415, 76416, 76417, 76418, 76421, 76422, 76423, 76424, 76425, 76426, 76427, 76428, 76491, 76492, 76493, 76494, 76495, 76496, 76497, 76498, 76491, 76492, 76493, 76494, 76495, 76496, 76497, 76498, 76501, 76502, 76503, 76504, 76505, 76506, 76507, 76508, 76511, 76512, 76513, 76514, 76515, 76516, 76517, 76518 or the ICD-10 codes of P0501, P0502, P0503, P0504, P0505, P0506, P0507, P0508, P0511, P0512, P0513, P0514, P0515, P0516, P0517, P0518, P070, P0700, P0701, P0702, P0703, P07014, P07015, P07016, P07017, P07018.

**Additional File 2**

**Details on Statistical Analysis**

The difference-in-differences model we estimate is:

$$\begin{aligned} Y_{i,t}=\beta_{0}+\beta_{1}{treated}_{i}+\beta_{2}{post}_{t}+\beta_{3}{post}_{t}*{treated}_{i}+\beta_{4}X_{i}+\gamma_{t}+\varepsilon_{i,t} \end{aligned}$$

where *i* denotes the infant and *t* is the year. $Y_{i,t}$ is an indicator for outcome variables. ${treated}_{i}$ is a binary variable, which takes value 1 if the patient resides in the Arkansas side of Texarkana, and 0 otherwise. ${post}_{t}$ is a binary variable, which takes value 1 if year>=2014, 0 otherwise. $X_{i}$ represents individual characteristics including race, sex, and dummies for patient zip codes, $\gamma_{t}$ includes year-quarter dummies, and $\varepsilon_{i,t}$ is the error term. $\beta_{3}$ is the parameter of interest and captures the overall difference-in-differences effect i.e., whether the change in the outcome variables is different for the treatment and control group. Technically adding zip code dummies with the treatment dummy and quarter dummies with the post dummy creates multicollinearity. In this case, treatment and post dummies would represent one zip code or one quarter after the Medicaid expansion and should be interpreted with caution. Therefore, we focus on the interaction of treatment and post dummies.

We estimate the difference-in-differences model for each racial/ethnicity group separately. We also estimate the following difference-in-difference-in-differences model to explore if the expansion affected different racial/ethnic groups differently:

$$\begin{aligned} Y_{i,t}=\beta_{0}+\beta_{1}{treated}_{i}+\beta_{2}{post}_{t}+\beta_{3}{race}_{i}+\beta_{4}{post}_{t}*{treated}_{i}+\beta_{5}{post}_{t}*{race}_{i}+ \\ \\ \beta_{6}{treated}_{i}*{race}_{i}+\beta_{7}{post}_{t}*{treated}_{i}*{race}_{i}+\beta_{8}Z_{i}+\gamma_{t}+ \sigma_{i,t} \# \end{aligned}$$

where ${race}_{i}$ is an indicator variable for non-Hispanic Black or Hispanic. The baseline group is non-Hispanic White. $Z_{i}$ includes sex and zip code dummies, $\gamma_{t}$ includes year-quarter dummies, and $\sigma_{i,t}$ is the error term. $\beta_{7}$ is the key parameter, which shows whether the treatment affected races differentially.

We employ a difference-in-differences event study framework to examine the gradual impact of the expansion on birth outcomes. We estimate the following equation:

$$Y_{i,t}=\alpha_{0}+\alpha_{1}{treated}_{i}+\sum_{t=2010}^{2012} \alpha_{2t}{year}_{t}+\sum_{t=2010}^{2012} \alpha_{3t}{year}_{t}*{treated}_{i}+\sum_{t=2014}^{2019} \alpha_{4t}{year}_{t}+\sum_{t=2014}^{2019} \alpha_{5t}{year}_{t}*{treated}_{i}+{\alpha_{6}X_{i}+\omega}_{i,t}$$

where $Y_{it}$ represents the outcome variables. ${treated}_{i}$ is an indicator for infants from Arkansas. ${year}_{t}$ represents year dummies. The year 2013 is omitted as the reference year. The coefficients of interest are $\alpha_{5t}$ that captures the difference between the outcome of the treated group and control group in each year (t) from 2014, the year of Medicaid expansion, to 2019.

**Parallel Trends Test**

The validity of the difference-in-differences analysis hinges on the assumption that the difference in birth outcomes between the infants from Texas and Arkansas would have remained constant over time if Medicaid expansion in Arkansas had not taken place (parallel trends assumption). We test the parallel trends assumption by estimating the following equation for the pre-treatment period from the first quarter of 2010 to the last quarter of 2013:

$$\begin{aligned} Y_{i,t}=\beta_{0}+\beta_{1}{treated}_{i}+\sum_{t=2010,1}^{2013,4} \beta_{2t}{yq}_{t}+\sum_{t=2010,1}^{2013,4} \beta_{3t}{yq}_{t}*{treated}_{i}+{\beta_{4}X_{i}+\vartheta}_{i,t} \end{aligned}$$

where $Y_{it}$ represents the outcome variables. ${treated}_{i}$ is an indicator for infants from Arkansas. ${yq}_{t}$ represents year-quarter dummies. The coefficients of interest are $\beta_{3t}$, which capture if outcome variables are different for the treated group in each period before Medicaid expansion. We also test if $\beta_{3t}=0, \forall t$, i.e., the coefficients of the interactions between treatment and year-quarter dummies are jointly significant. Failure to reject the null hypothesis provides evidence that the parallel trends assumption is not violated. We report the interaction coefficients and the p-values associated with the joint significance test. Supplemental Table 1 shows the regression results. We fail to reject that the time trends for birth outcomes of newborns from Texas and Arkansas sides of Texarkana were different in the pre-expansion period at the 5% level. Hence, we do not reject that the parallel trends assumption holds.

Supplemental Table 1: Adjusted parallel trends

|  | Overall | | White | | Black | | Hispanic | |
| --- | --- | --- | --- | --- | --- | --- | --- | --- |
|  | Preterm | Low bw | Preterm | Low bw | Preterm | Low bw | Preterm | Low bw |
| 2010q2*treated | -3.38 | -3.60 | -0.96 | -2.10 | -12.02* | -10.83* | 7.78 | 10.01 |
|  | (-8.98, 2.22) | (-8.30, 1.11) | (-7.80, 5.88) | (-7.27, 3.07) | (-24.23, 0.18) | (-22.75, 1.10) | (-13.43, 28.98) | (-7.75, 27.77) |
| 2010q3*treated | 0.89 | -0.57 | 5.27 | 2.38 | -9.43 | -6.06 | 7.39 | 5.51 |
|  | (-4.68, 6.47) | (-5.26, 4.11) | (-1.63, 12.17) | (-2.83, 7.60) | (-21.45, 2.60) | (-17.82, 5.69) | (-9.74, 24.51) | (-8.83, 19.85) |
| 2010q4*treated | -0.92 | -2.99 | 1.79 | -3.39 | -7.20 | -2.09 | 7.26 | 5.93 |
|  | (-6.60, 4.77) | (-7.77, 1.78) | (-5.28, 8.85) | (-8.73, 1.95) | (-19.30, 4.89) | (-13.91, 9.72) | (-10.63, 25.16) | (-9.06, 20.91) |
| 2011q1*treated | -4.62 | -5.35** | -3.31 | -4.74* | -11.54* | -10.89* | 13.24 | 11.00 |
|  | (-10.36 - 1.12) | (-10.17, -0.53) | (-10.33, 3.72) | (-10.06, 0.57) | (-23.96, 0.89) | (-23.04, 1.25) | (-5.82, 32.30) | (-4.96, 26.96) |
| 2011q2*treated | -3.43 | -3.07 | -2.72 | -1.70 | -12.12* | -11.72* | 8.28 | 6.62 |
|  | (-9.03 - 2.17) | (-7.78, 1.63) | (-9.84, 4.40) | (-7.09, 3.69) | (-24.60, 0.35) | (-23.91, 0.47) | (-8.45, 25.01) | (-7.39, 20.63) |
| 2011q3*treated | -1.40 | -3.84 | 1.60 | -0.82 | -1.97 | -6.10 | 6.74 | 8.70 |
|  | (-7.05 - 4.24) | (-8.58, 0.90) | (-6.43, 9.64) | (-6.89, 5.26) | (-13.99, 10.05) | (-17.85, 5.65) | (-12.36, 25.84) | (-7.30, 24.69) |
| 2011q4*treated | -5.32* | -5.10** | 2.15 | -1.32 | -15.85*** | -12.80** |  |  |
|  | (-10.71, 0.08) | (-9.64, -0.57) | (-5.57, 9.87) | (-7.16, 4.51) | (-27.52, -4.19) | (-24.19, -1.40) |  |  |
| 2012q1*treated | -6.60** | -3.39 | -7.19* | -3.19 | -9.12 | -3.58 | 3.38 | 2.59 |
|  | (-12.23, -0.97) | (-8.11, 1.34) | (-14.78, 0.41) | (-8.94, 2.55) | (-21.06, 2.81) | (-15.25, 8.09) | (-24.64, 31.39) | (-20.88, 26.05) |
| 2012q2*treated | 0.65 | 0.53 | 3.69 | 2.67 | -2.21 | -4.01 | -5.47 | 1.97 |
|  | (-4.84, 6.14) | (-4.08, 5.15) | (-3.51, 10.90) | (-2.78, 8.11) | (-14.19, 9.77) | (-15.71, 7.70) | (-25.91, 14.96) | (-15.15, 19.08) |
| 2012q3*treated | -3.49 | -3.13 | -4.58 | -3.13 | -5.20 | -4.93 | -3.10 | -1.25 |
|  | (-8.95, 1.96) | (-7.71, 1.45) | (-11.80, 2.63) | (-8.58, 2.33) | (-16.67, 6.28) | (-16.14, 6.29) | (-23.16, 16.96) | (-18.04, 15.55) |
| 2012q4*treated | -1.78 | -1.92 | -2.19 | -2.46 | -8.53 | -7.69 | 14.00 | 10.87 |
|  | (-7.21, 3.65) | (-6.48, 2.64) | (-9.45, 5.07) | (-7.95, 3.03) | (-20.11, 3.05) | (-19.00, 3.63) | (-8.08, 36.08) | (-7.62, 29.36) |
| 2013q1*treated | -0.80 | -2.10 | -1.56 | -0.47 | -5.24 | -7.36 | 11.16 | 13.60 |
|  | (-6.29, 4.69) | (-6.71, 2.51) | (-8.86, 5.74) | (-5.99, 5.06) | (-16.83, 6.35) | (-18.68, 3.97) | (-12.63, 34.95) | (-6.33, 33.52) |
| 2013q2*treated | -2.28 | -2.35 | 1.33 | 0.08 | -12.95** | -10.60* | -5.34 | 5.22 |
|  | (-7.93, 3.38) | (-7.10, 2.40) | (-5.54, 8.20) | (-5.11, 5.28) | (-25.31, -0.60) | (-22.67, 1.47) | (-25.95, 15.26) | (-12.04, 22.47) |
| 2013q3*treated | 0.46 | 1.16 | 1.78 | 1.96 | -2.67 | 0.11 | 6.07 | 7.55 |
|  | (-5.01, 5.92) | (-3.43, 5.75) | (-4.91, 8.48) | (-3.10, 7.03) | (-14.26, 8.91) | (-11.21, 11.42) | (-15.74, 27.88) | (-10.72, 25.82) |
| 2013q4*treated | -0.59 | 0.52 | 0.92 | 1.58 | -5.75 | -3.16 | 11.29 | 6.92 |
|  | (-6.01, 4.82) | (-4.03, 5.07) | (-5.75, 7.58) | (-3.46, 6.62) | (-17.55, 6.05) | (-14.69, 8.37) | (-6.99, 29.57) | (-8.39, 22.23) |
|  |  |  |  |  |  |  |  |  |
| P(F-statistics) | 0.20 | 0.10 | 0.13 | 0.17 | 0.27 | 0.44 | 0.81 | 0.98 |
| Observations | 10,233 | 10,233 | 5,528 | 5,528 | 2,743 | 2,743 | 644 | 644 |
|  |  |  |  |  |  |  |  |  |

Notes: 95 % confidence intervals in parentheses. bw: birth weight. P(F-statistics) is the p-value for the F statistics that tests joint significance of the interaction coefficients. The reference period is year 2010, quarter 1. Coefficients for 2011q4*treated are not reported for birth outcomes for Hispanic births due to lack of observations in that quarter. *** p<0.01, ** p<0.05, * p<0.1 The table reports the estimates of $\beta_{3t}, \forall t$ obtained from regressions adjusted for sex, race/ethnicity, year-quarter, and patient zip code dummies.

Supplemental Figure 1 plots unadjusted averages of outcome variables overall and by race/ethnicity. We omitted the Hispanic group due to the lack of observations in some quarters.

**Supplemental Figure 1:** Unadjusted averages of birth outcomes over time

| 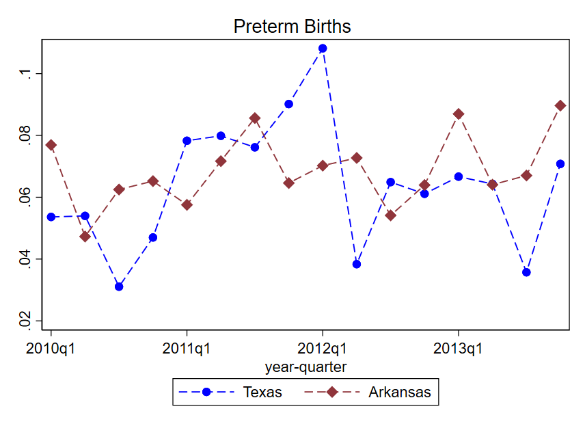 | 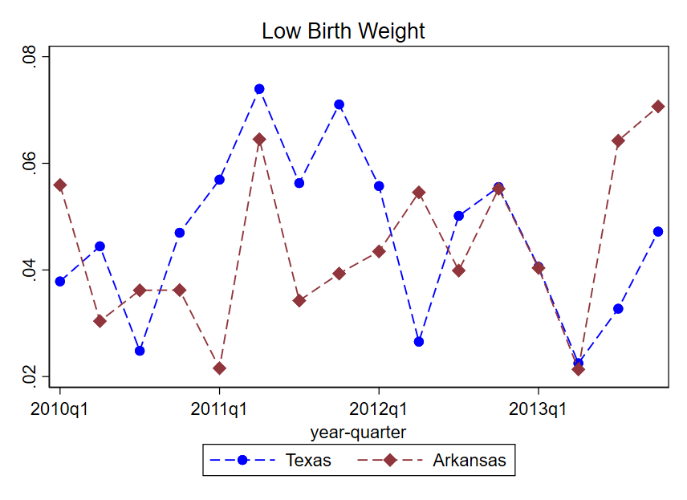 |
| --- | --- |
| 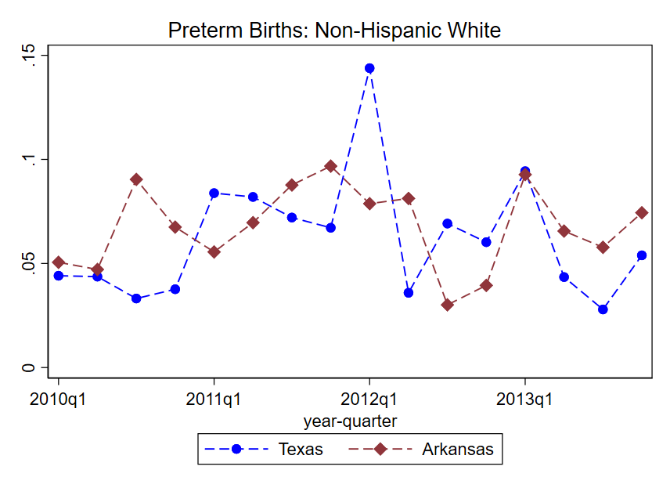 | 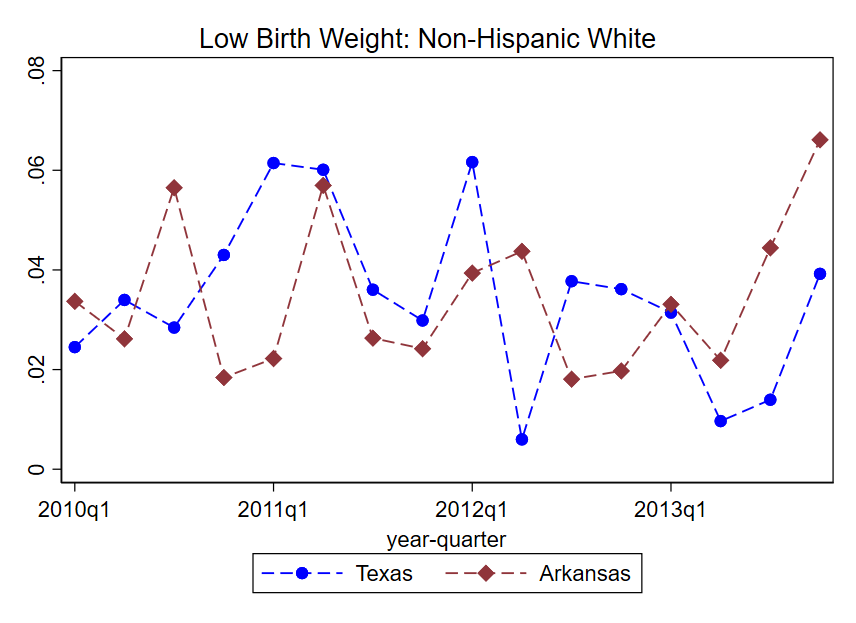 |
| 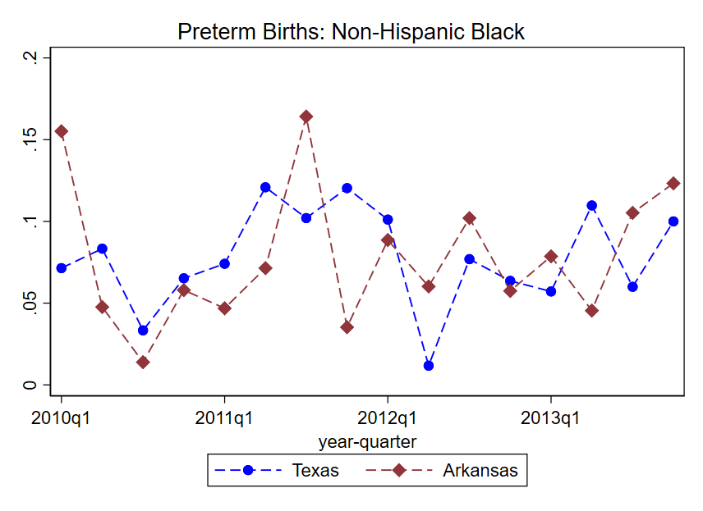 | 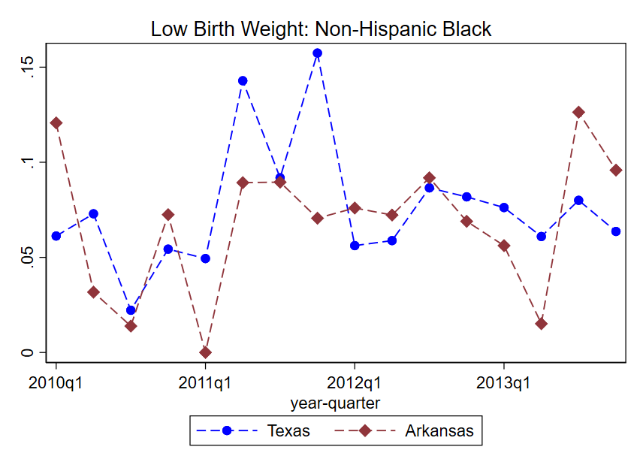 |

**Additional File 3**

Supplemental Table 2: Unadjusted and adjusted changes in mothers’ comorbidities associated with Medicaid expansion

|  | **Arkansas** | | **Texas** | | **DID** | | **DDD** |
| --- | --- | --- | --- | --- | --- | --- | --- |
|  | **Pre-** | **Post-** | **Pre-** | **Post-** | **Unadjusted (95% CI)** | **Adjusted (95% CI)** | **Adjusted (95% CI)** |
| **Overall** |  |  |  |  |  |  |  |
| Charlson index | 0.0206 | 0.0318 | 0.0243 | 0.0368 | -0.0014 (-0.011, 0.008) | -0.0035 (-0.013, 0.006) |  |
| **Non-Hispanic White** |  |  |  |  |  |  |  |
| Charlson index | 0.0204 | .0207 | 0.0226 | 0.0322 | -0.0094 (-0.022, 0.003) | -0.0085 (-0.021, 0.004) |  |
| **Non-Hispanic Black** |  |  |  |  |  |  |  |
| Charlson index | 0.0270 | 0.0453 | 0.0332 | 0.0441 | 0.0075 (-0.012, 0.027) | 0.0017 (-0.017, 0.020) | 0.0112 (-0.011, 0.033) |

*Notes:* Pre-, Pre-expansion period (2010-2013); Post-, Post-expansion period (2014-2019); DID, difference-in-differences; DDD, difference-in-difference-in-differences; CI, confidence interval. In the DDD regression, non-Hispanic Whites are the baseline group. Adjusted coefficients are from regressions controlling for year-quarter, and patient zip code dummies. Dummies for race/ethnicity categories are also included for the overall results.

We have examined the child delivery records to identify the possible causes for failing to find a statistically significant association between Medicaid expansion and birth outcomes for Black infants. In our dataset, the share of Black mothers residing in Arkansas increased from 25.4% to 28.2% post-expansion, whereas the share of Black mothers residing in Texas slightly declined from 30.4% to 30.1%. Even though we cannot match newborns with mothers, we used child delivery records to examine if there have been any differences in terms of average comorbidities (measured by Charlson Comorbidity Index) of mothers giving birth before and after the expansion by state and by race/ethnicity. Supplemental Table 2 reports the average Charlson Comorbidity Indices of mothers giving birth in Arkansas and Texas sides of Texarkana in the pre-expansion and post-expansion periods, difference-in-differences, and difference-in-difference-in-differences regression results with Charlson Comorbidity Index being the dependent variable. Charlson Comorbidity Index of Black and White mothers increased by 0.01 for both Black and White mothers in Texas from pre-expansion to post-expansion. While average Charlson Comorbidity Index was 0.03 for Black mothers and 0.02 for White mothers in Arkansas in the pre-expansion period, it increased to 0.05 for Black mothers and remained at 0.02 for White mothers in the post-expansion period. Even though the difference-in-differences and difference-in-difference-in-differences estimates lack statistical power, the average comorbidity index for Black mothers has risen in Arkansas compared to those in Texas, which suggests that our failure to find a statistically significant improvement for Black infants maybe due to a higher share of Black mothers with comorbidities who might not have been able to conceive and continue their pregnancy if Medicaid had not been expanded.

Supplemental Table 3: Change in age distribution (%)

|  | **Arkansas** | | | **Texas** | | |
| --- | --- | --- | --- | --- | --- | --- |
|  | **Pre** | **Post** | **Post-Pre** | **Pre** | **Post** | **Post-Pre** |
| **Non-Hispanic White** | | | | | | |
| <20 | 12.51 | 9.33 | -3.19*** | 11.84 | 9.40 | -2.43*** |
| 20-34 | 80.18 | 82.90 | 2.73*** | 80.54 | 80.77 | 0.23 |
| 35+ | 6.74 | 7.06 | 0.32 | 7.00 | 8.86 | 1.86*** |
| HIV or Alcohol/Drug use | 0.56 | 0.71 | 0.63 | 0.63 | 0.96 | 0.34 |
| **Non-Hispanic Black** | | | | | | |
| <20 | 17.26 | 14.96 | -2.29* | 18.89 | 15.03 | -3.86*** |
| 20-34 | 77.08 | 78.96 | 1.88 | 74.08 | 77.27 | 3.19** |
| 35+ | 4.71 | 4.80 | 0.09 | 5.80 | 6.56 | 0.77 |
| HIV or Alcohol/Drug use | 0.95 | 1.28 | 0.32 | 1.25 | 1.14 | -0.10 |

*Notes:* Pre-post difference is based on a simple mean test (two-sided t-test). *** p<0.01, ** p<0.05, *p<0.1. Patients with HIV or drug/alcohol use are put in wider age categories for privacy concerns. That group includes patients between ages 18-44.

Supplemental Table 3 shows the distribution of childbirth patients into different age categories in both states, before and after Medicaid expansion. For White mothers, the share of teenage pregnancies declined in both Arkansas and Texas from the pre-expansion period to the post-expansion period, with a larger decline in Arkansas than in Texas. The share of White mothers with age 20-34 increased whereas there was no statistically significant change in the share of mothers with age 35 and over from the pre-expansion period to the post-expansion period in Arkansas, while the share of White mothers with age 35 and over increased from pre-expansion period to post-expansion period in Texas. For Black mothers, the share of teenage pregnancies declined from the pre-expansion period to the post-expansion period in both Arkansas and Texas, with a larger decline in Texas than that of Arkansas. There was no statistically significant change in the share of Black mothers aged 20-34 from the pre-expansion period to the post-expansion period in Arkansas, while this share increased in Texas.

Supplemental Table 4: Change in payer types (%)

|  | **Arkansas** | | | **Texas** | | |
| --- | --- | --- | --- | --- | --- | --- |
|  | **Pre** | **Post** | **Post-Pre** | **Pre** | **Post** | **Post-Pre** |
| **Non-Hispanic White** | | | | | | |
| Medicaid | 36.14 | 35.06 | -1.8 | 40.21 | 43.28 | 3.06** |
| Private | 59.09 | 61.78 | 2.68** | 57.73 | 54.73 | -3.00** |
| Uninsured | 4.76 | 3.17 | -1.60*** | 2.05 | 2.00 | 0.07 |
| **Non-Hispanic Black** | | | | | | |
| Medicaid | 62.62 | 61.22 | -1.40 | 73.37 | 75.23 | 1.86 |
| Private | 36.00 | 37.36 | 1.39 | 25.52 | 24.00 | -1.53 |
| Uninsured | 1.39 | 1.40 | 0.01 | 1.10 | 0.78 | -0.33 |

*Notes:* Pre-post difference is based on a simple mean test (two-sided t-test). *** p<0.01, ** p<0.05

Supplemental Table 4 shows the share of different payer types for childbirth patients in both states before and after the Medicaid expansion. There were no significant changes in the payer type for Non-Hispanic Blacks before and after the expansion in both states. In Arkansas, the uninsurance rate decreased significantly while the share of privately insured increased for Whites. Given that Arkansas expanded Medicaid through private option, these numbers suggest that non-Hispanic whites benefited from increased insurance coverage. In Texas, the increase in Medicaid is matched by an equivalent decrease in private insurance with no significant change in the uninsurance rate.
